# Myoepithelial Cells: Any role in aspiration cytology smears of breast tumors?

**DOI:** 10.1186/1742-6413-5-9

**Published:** 2008-04-21

**Authors:** Sanjib Kumar Pattari, Pranab Dey, Subhash K Gupta, Kusum Joshi

**Affiliations:** 1 Department of Pathology, Post Graduate Institute of Medical Education and Research, Chandigarh, India; 2 Department of Cytology, Post Graduate Institute of Medical Education and Research, Chandigarh, India; 3 Department of Histopathology, Post Graduate Institute of Medical Education and Research, Chandigarh, India

## Abstract

**Aims and Objective:**

To study the role of myoepithelial (ME) cells in distinguishing benign, proliferative breast diseases (PBD) and frank malignant breast lesions.

**Materials and methods:**

In this study, histology proven 71 cases of fine needle aspiration cytology (FNAC) of palpable breast lesions were selected. There were 30 invasive carcinomas (24 infiltrating duct carcinoma and 6 infiltrating lobular carcinoma), 25 cases of benign lesion (21 fibroadenomas and 4 fibrocystic lesions) and 11 proliferative breast diseases (other than carcinoma in situ) and five cases of carcinoma in situ. The number of ME cells were estimated in respect to 1000 ductal cells. In every case at least 20 high power fields (× 40) were studied. Quantitative estimation of ME cell was correlated with the final diagnosis. Corresponding histopathology cases were also evaluated for diagnostic confirmation along with the pattern of distribution of ME cells. The ME cells were also quantitated on histopathology sections on smooth muscle actin (SMA) immunostained sections.

**Results:**

The mean number of ME cells per 1000 ductal cells on cytology smears was 5.1 ± 5.5, 30.8 ± 25, 28.3 ± 20.2, and 38.4 ± 38.8 in malignant, carcinoma in situ, PBD and benign breast lesions respectively. The non parametric Mann Whitney test showed significant difference in number of the ME cells between benign and malignant groups (p < .000), PBD and malignant groups (p < .000) and carcinoma in situ and malignant group (p < .001). However, it was insignificant between benign and PBD group, and PBD and carcinoma in situ (p > .01). In SMA stained histopathology sections, ME cell in benign, PBD, carcinoma in situ and malignant cases were 741.12 ± 248, 238 ± 172, 121.6 ± 115 and 15.6 ± 25.1 respectively. Statistical analysis showed significantly different number of ME cell between benign versus PBD group, carcinoma in situ and malignant group. It was also significant between PBD versus malignant, and carcinoma in situ versus malignant (p < .001, Mann Whitney test). However number of ME cell was not significant between PBD versus carcinoma in situ.

**Conclusion:**

The number of ME cell in breast lesions may be helpful in distinguishing PBD versus invasive malignant tumors on FNAC smears. However it is not helpful to distinguish benign lesions versus PBD.

## Background

Fine needle aspiration cytology (FNAC) is an important tool for rapid and accurate diagnosis of various benign and malignant breast lesions with high sensitivity and specificity [1-4]. However in certain occasion it is difficult to provide a definitive diagnosis in breast neoplasm with the help of FNAC. This is particularly true in proliferative breast diseases (PBD), and certain types of carcinomas such as tubular carcinoma and lobular carcinomas [5-7]. The presence of myoepithelial cell (ME) has long been recognized as a prominent feature of benign breast diseases [8]. However its presence in proliferative breast diseases has rarely been explored particularly in FNAC smears [8-11]. In this present paper we studied the role of quantitation of ME cells in distinguishing benign, PBD and frank malignant breast lesions.

## Materials and methods

In this study, histology proven 71 cases of FNAC of palpable breast lesions were selected. There were 30 invasive malignant tumors (24 cases of infiltrating duct carcinoma and 6 cases of infiltrating lobular carcinoma), 25 cases of benign lesion (21 fibroadenomas and 4 fibrocystic lesions) and 11 proliferative breast diseases and five cases of carcinoma in situ (CIS). Both malignant and benign breast lesions were selected randomly. FNAC smears of all the histopathology proven PBD cases diagnosed in last four years were selected for this study. The PBD cases were classified as described by Page DL et al [6]. The carcinoma in situ cases were classified as described by Holland R et al [12]. In all the cases both May Grunwald Giemsa and haematoxylin and eosin stained smears were studied. The ME cells in FNAC smears were identified as oval to bipolar cells with scanty cytoplasm and elongated densely stained nuclei. The number of ME cells were estimated in respect to 1000 ductal cells. In every case at least 20 high power fields (× 40) were studied. Quantitative estimation of ME cell was correlated with the final diagnosis. Corresponding histopathology cases were also evaluated for diagnostic confirmation along with the pattern of distribution and counting of ME cells on smooth muscle actin (SMA) immunostained sections (DAKO muscle actin, HHF 35).

## Results

The distribution of cases was shown in table 1. In group A, there were 25 benign breast lesions comprised of 21 cases of fibroadenoma (FA) and four cases of fibrocystic diseases. In group B, there were 11 cases of PBD comprised of mild hyperplasia (5), moderate hyperplasia (4), and atypical ductal hyperplasia (2). In group C there were five cases of carcinoma in situ (5), out of which three were three high grade and one each intermediate grade and low grade tumor. In group D, there were 30 malignant tumors comprised of 24 cases of infiltrating duct carcinoma and 6 cases of infiltrating lobular carcinoma.

**Table 1:** Distribution of cases

**Diagnosis**	**Number of cases**
**Diagnosis**	**Number of cases**
Fibroadenoma	21
Fibrocystic cases	4
**Group B, Proliferative breast disease**	
Mild Hyperplasia	5
Moderate Hyperplasia	4
Atypical ductal Hyperplasia	2
**Group C, Carcinoma in situ**	
Carcinoma in situ	5
**Group D, Invasive malignant tumor**	
Infiltrating duct carcinoma	24
Infiltrating lobular carcinoma	6

**Figure 1:**
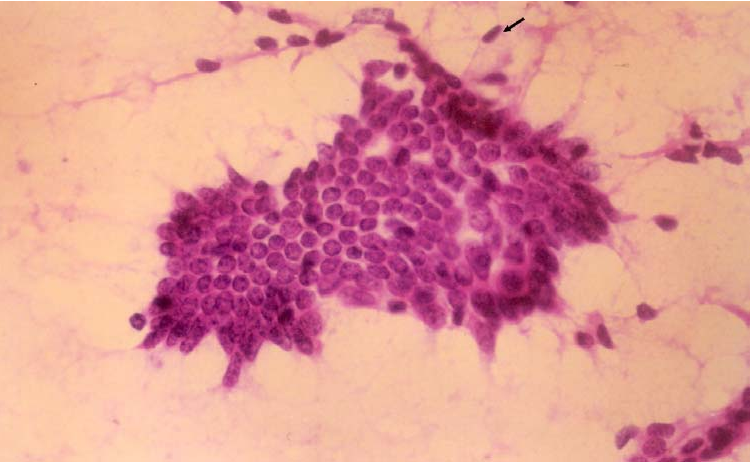
Many scattered myoepithelial cells in cytology smear of fibroadenoma. (Haematoxylin and Eosin × 480).

**Figure 2:**
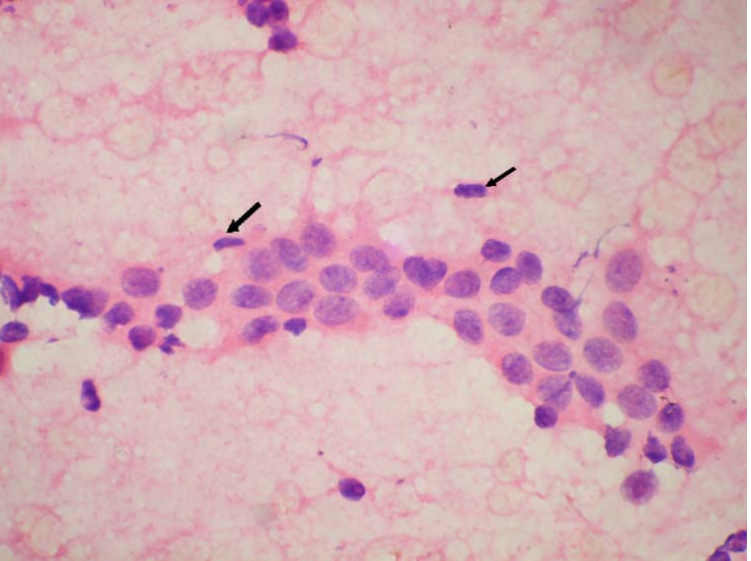
Occasional myoepithelial cells in cytology smear of moderate hyperplasia. (Haematoxylin and Eosin × 480).

**Figure 3:**
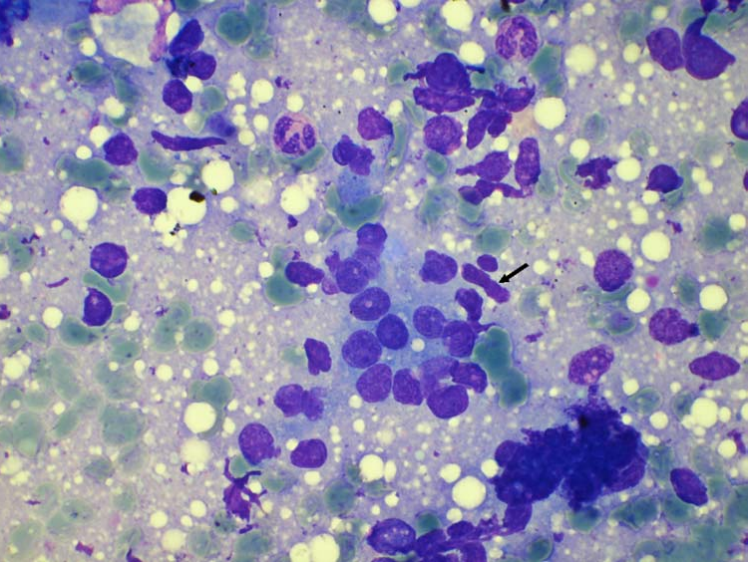
Myoepithelial cells in cytology smear of carcinoma in situ. (May Grunwald Giemsa stain × 480).

The ME cells were distributed as scattered singly in the background in between the epithelial cell clusters and within the epithelial cell clusters (figure 1, 2, 3). The mean number of ME cells per 1000 ductal cells on cytology smears was 5.1 ± 5.5, 30.8 ± 25, 28.3 ± 20.2, and 38.4 ± 38.8 in malignant, carcinoma in situ, PBD and benign breast lesions respectively (Table 2). The non parametric Mann Whitney test showed significant difference in number of the ME cells between benign and malignant groups (p < .000), PBD and malignant groups (p < .000) and carcinoma in situ and malignant group (p < .001). However, the number of the ME cells was insignificant between benign and PBD group, and PBD and carcinoma in situ (p > .01).

**Table 2:** Mean number of myoepithelial cells in cytology smear

**Diagnostic groups**	**Number of cases**	**Mean**	**Standard deviation (±)**
**Group A, Benign**	25	38.4	38.8
**Group B, Proliferative breast disease**	11	28.3	20.2
**Group C, Carcinoma in situ**	5	30.8	25.0
**Group D, Invasive malignant tumor**	30	5.1	5.5

Mann Whitney's U Test

Group A and Group B, p = 0.685, not significant

Group A and Group C, p = 0.706, not significant

Group A and Group D, p = 0.000, significant

Group B and Group C, P = 0.913, not significant

Group B and Group D, p = .000, significant

Group C and Group D, p = 0.001, significant

**Figure 4:**
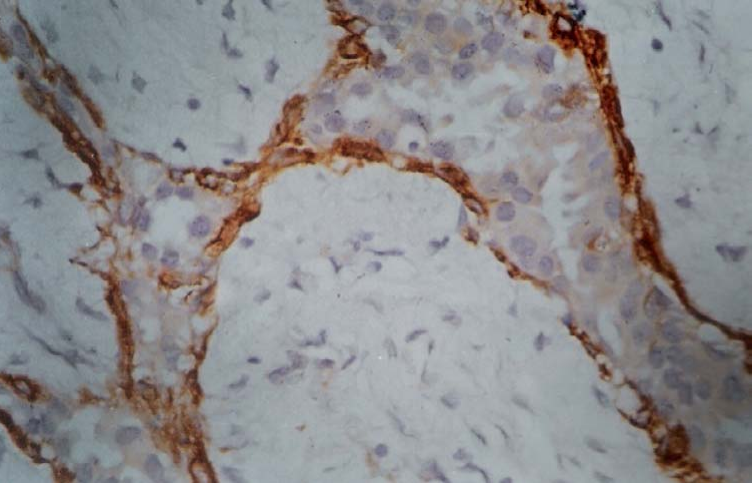
Circumferential presence of myoepithelial cells in SMA stained histology section of fibroadenoma. (Smooth muscle actin × 480).

**Figure 5:**
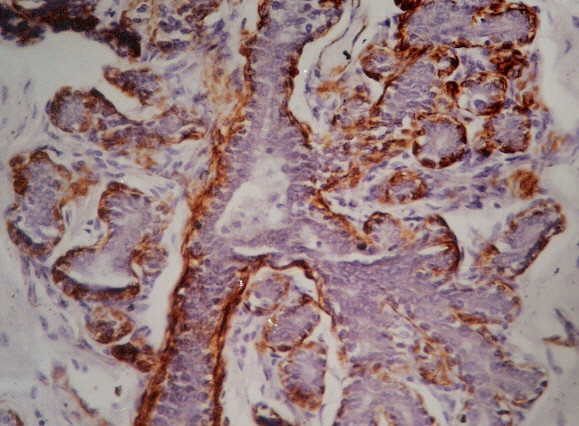
Myoepithelial cells in SMA stained histology section of mild hyperplasia. (Smooth muscle actin × 480).

The SMA staining gave consistent staining of ME cells, which were distributed as a continuous circumferential layer around the ducts and acini of normal breast tissue. The uniformly circumferential staining pattern was maintained in FA, which however became compressed in ducts with marked invagination due to stromal connective tissue overgrowth (figure 4). In PBD, the circumferential pattern of staining was mostly maintained. The uniformity of distribution and number of stained cells varied depending upon the histological spectrum of the lesion. In mild or moderate PBD, the staining was uniformly circumferential (figure 5). In carcinoma in situ, the affected ducts and acini showed interrupted pattern of staining with marked thinning. In IDC, there was almost complete absence of SMA stained cells around the tumor cells. In smooth muscle actin stained histopathology sections, ME cell in benign, PBD, carcinoma in situ and malignant cases were 741.12 ± 248, 238 ± 172, 121.6 ± 115 and 15.6 ± 25.1 respectively (Table 3). Statistical analysis showed significantly different number of ME cell between benign versus PBD group, carcinoma in situ and malignant group (Mann Whitney test). It was also significant between PBD versus malignant, and carcinoma in situ versus malignant (p < .001, Mann Whitney test). However number of ME cell was not significantly different between PBD versus carcinoma in situ.

**Table 3:** Mean number of myoepithelial cells in SMA stained histology sections

**Diagnostic groups**	**Number of cases**	**Mean**	**Standard deviation (±)**
**Group A, Benign**	25	741.12	248
**Group B, Proliferative breast disease**	11	238	172
**Group C, Carcinoma in situ**	5	121.6	115
**Group D, Invasive malignant tumor**	30	15.6	25.1

Mann Whitney's U Test

Group A and Group B, p = 0.000, significant

Group A and Group C, p = 0.000, significant

Group A and Group D, p = 0.000, significant

Group B and Group C, P = 0.234, not significant

Group B and Group D, p = .000, significant

Group C and Group D, p = 0.000, significant

## Discussion

Ductal system of breast is composed of epithelial cells, ME cells and intermediate or basal clear cells. In FNAC smears, ME cells are identified as oval to elongated bipolar cells with stripped of cytoplasm. In this present study, the number of ME cells in FNAC smears was significantly decreased between benign and malignant lesions as well as between PBD and malignant lesions, but the difference was not significant between benign and PBD. Yu et al [8] also quantified the number of ME cells per 40 high power field and showed that single ME cell could be seen in both benign and malignant lesions. They noted that paired ME cells was a feature of benign lesions only and this was not present in malignant lesions. In the present study, pairing of ME cell was not a significant observation even in benign lesion.

Masood S et al [13] demonstrated significant difference of number of ME cell in benign versus malignant tumors. Unlike this study, we compared the number of ME cell between PBD with benign and malignant lesions.

Bofin AM et al [10] compared the cytologic features of benign PBD, DCIS and invasive carcinoma on FNAC smears. They noted that nuclear morphology, myoepithelial cells, signs of invasion and degree of cellular differentiation are the most important discriminating factors to distinguish between different groups of lesions. Similar to the present study they also noted that ME cells are virtually absent or markedly reduced in invasive carcinoma compared to DCIS lesions. Bofin AM et al only labeled the presence or absence of ME cells. However we tried to quantify the number of such cells on FNAC smears [10].

The number of ME cell was helpful in distinguishing PBD and malignant lesions but not between PBD and benign lesion. In histopathology sections stained by SMA, the number of ME cell was significantly different between benign and PBD, benign and malignant; as well as between PBD and malignant lesions. Thus ME cell differentiation is present in benign and proliferative disorders. However, this differentiation is lost in carcinoma [14]. In histopathology, a statistically significant difference in ME cell number was noted between benign and PBD, However, in cytology this difference was not statistically significant. This may be because of the limitation of proper sampling from the representative area in FNAC smears. It was easier to select out the representative areas for counting, whereas this was not possible in FNAC smears.

Confirmation of ME cells on routine cytology or histology can be done with the help of immunostaining. We performed SMA immunostaining. However these cells can also be identified by S-100[15], calponin [16], h-caldesmin[16,17], smooth muscle heavy chain (SMMHC) antibodies [16,17] and CD10 [18]. Foschini MP et al [16] showed that SMMHC is more specific for ME cells in breast.

Atypical ductal hyperplasia is a pre-malignant lesion and carcinoma in situ is considered as pre-invasive malignant lesion [19]. It is of great clinical importance to identify accurately these lesions on FNAC. The number of ME cells may be helpful in distinguishing between PBD versus DCIS and invasive carcinoma on FNAC smears. However, in routine reporting, it is not possible to quantitate the number of ME cells and probably a subjective assessment could be done along with other cytology features.

## Competing interests

The author(s) declares that they have no competing interests.

## Authors' contributions

SP collected data, analyzed it and partially drafted the manuscript. PD designed, analyzed and did final drafting of the manuscript. SKG was involved in conception and designing of the study, and interpretation of data. KJ was involved in data retrieval and analysis.

All authors read and approved the final manuscript.
